# YOLOv11-SMS: An Improved Algorithm for Impurity Detection in Seed Cotton

**DOI:** 10.3390/s26092835

**Published:** 2026-05-01

**Authors:** Wenyan Yuan, Laigang Zhang, Donghe Wang, Zhijun Guo

**Affiliations:** 1 School of Mechanical and Automotive Engineering, Liaocheng University, Liaocheng 252000, China; 2 Changchun Institute of Optics, Fine Mechanics and Physics, Chinese Academy of Sciences, Changchun 130033, China; 3 Institute of Information Science and Technology, Hunan Normal University, Changsha 410081, China

**Keywords:** seed cotton impurity detection, YOLOv11, LRSA, MBRConv, SAFM

## Abstract

To enhance the precision of cottonseed impurity detection and address issues such as high miss-detection rates and suboptimal performance, this paper introduces an improved YOLOv11 algorithm, termed YOLOv11-SMS. Initially, the algorithm integrates a local self-attention mechanism (LRSA) to design the C2PSA-SL module, which augments the model’s ability to learn local information while maintaining global feature awareness. Furthermore, the feature extraction stage and the network head incorporate a multi-branch reparameterized convolution (MBRConv) module, enhancing feature extraction capabilities while preserving the model’s lightweight properties. Lastly, a spatial adaptive modulation (SAFM) module is introduced to optimize the detection of small targets. Experimental results demonstrate that YOLOv11-SMS outperforms the baseline model, with mAP@_50–95_ increasing from 79.42% to 82.49%, an improvement of 3.07 percentage points. The average mIOU increased from 90.98% to 94.18%, representing a 3.2 percentage point improvement. Moreover, the model achieves an impressive real-time inference speed of 178.63 frames per second (FPS), effectively balancing detection accuracy and speed, offering an efficient and precise solution for cottonseed impurity detection.

## 1. Introduction

Cotton is one of China’s key agricultural crops and serves as the foundational raw material for the textile industry. It directly influences the quality, production efficiency, and market value of textiles, playing a crucial role in the coordinated development of both the agricultural and textile sectors in China. Cottonseed, the initial form of cotton after harvesting, inevitably contains impurities such as leaves, plastic film, and sand due to various stages including cultivation, mechanized harvesting, transportation, and preprocessing. The presence of these impurities has become a significant bottleneck limiting the improvement of the quality of China’s cotton industry.

Currently, traditional methods for detecting cottonseed impurities primarily rely on manual sorting. However, this approach suffers from low efficiency, high subjectivity, and time consumption, and no longer meets the demands of the modern cotton industry for large-scale, intelligent, and rapid detection. In recent years, with the rapid advancement of machine vision and deep learning technologies, object detection [1] techniques have provided new solutions for cottonseed impurity detection [2]. As representative one-stage detectors, the latest YOLO series, particularly YOLOv11, has demonstrated state-of-the-art performance and significant recent advances in small-target and multi-object detection tasks. These advancements, such as improved precision in small-target detection and faster inference times, have made YOLO models increasingly effective in applications such as seed cotton impurity detection [3,4,5,6]. YOLOv11′s ability to process complex visual information with high accuracy and speed makes it an ideal tool for real-time, large-scale detection tasks. This progress provides a solid foundation for leveraging and further improving YOLOv11 in the context of this study, effectively promoting the intelligent and automated transformation of the cotton industry.

In the case of seed cotton impurity detection, the core research object of this study, although several related studies have been reported, many challenges still remain. The main difficulties include the uneven fluffiness of the cotton layer, the high similarity between small impurities (such as paper, threads, films, etc.) and cotton texture, and the difficulty in promptly identifying and removing foreign fibers such as plastic film that are easily mixed in during the harvesting process. Additionally, poor field applicability remains a major problem in existing research, with the accuracy and robustness of detection models in complex environments (such as varying light intensity, different cotton varieties, and mixed impurities) requiring significant improvement. Therefore, designing a rapid, robust, and practically applicable intelligent detection method for small impurities in seed cotton is crucial, as it would not only enhance the cotton processing level and standardize the purchasing process in China but also contribute to the modernization of the country’s cotton industry.

Therefore, this paper proposes an improved method based on YOLOv11, termed YOLOv11-SMS. Experimental results demonstrate that this approach not only enhances the detection of impurities in cottonseed but also exhibits strong robustness and reliability.

## 2. Literature Review

Prior to the widespread adoption of deep learning [7,8], traditional object detection methods relied on manually designed features and machine learning classifiers for target recognition, suffering from poor robustness, limited generalization ability, low efficiency, and poor real-time performance. The advent of deep learning has offered a more efficient alternative, particularly for detecting small [9,10] and multiple [11,12] targets like cottonseed impurities in complex environments [13,14], significantly reducing false positives and missed detections.

To address the persistent challenges of low detection accuracy for small objects and the difficulty in balancing model complexity with real-time performance in cotton processing, researchers have explored various solutions. For example, Liu et al. [15] tackled the inefficiency and low accuracy of traditional cottonseed damage detection methods, as well as the slow inference speed, missed detections, and false positives of the original YOLOv5s model when dealing with small targets. They proposed an improved YOLOv5s model that replaces the Focus module with Denseblock, incorporates coordinate attention, adopts GhostConv for a more efficient architecture, removes the large-object detection layer, and employs the CIoU loss function. This enhanced model achieved a mean average precision (mAP@_50_) of 98.1% and a detection speed of 97 frames per second for two-category cottonseed classification. However, the study was limited to two cottonseed varieties and lacked validation across different cotton types and real-world industrial environments. He et al. [16] designed a compact Swin Transformer–YOLOv5n detector combined with an enhanced two-stage classification method to enable fine-grained cottonseed damage assessment, covering 4 major categories and 14 subcategories. The model reduces the parameter size by 30.11%, improves mAP@_50–95_ by 7.7% compared with YOLOv5n, and achieves a classification accuracy of 97.34%. Nevertheless, this approach still faces challenges such as class imbalance and missed detection of overlapping seeds. Gao et al. [17] presented a comprehensive review of machine vision-based methods for detecting foreign fibers in cotton, discussing various imaging techniques including X-ray, ultraviolet fluorescence, laser, and hyperspectral imaging, and tracing the evolution from traditional image processing to machine learning and deep learning models. They identified key issues such as insufficient real-time capability, the absence of standardized datasets, and difficulties in multi-modal fusion, and proposed future research directions focusing on multi-technology integration, lightweight algorithm development, and hardware–software co-design. Wang et al. [18] proposed a lightweight YOLO model incorporating the Swin Transformer to address the difficulty of tiny defect detection in complex environments. By effectively enhancing feature representation for small objects, this method improves both precision and recall, delivering more stable and accurate tiny defect detection results under challenging conditions.

To address the difficulties in identifying white and transparent foreign fibers as well as the low detection accuracy of multi-size mixed impurities in seed cotton, researchers have explored methods combining hyperspectral imaging technology with deep learning, achieving remarkable progress in cotton quality detection and impurity recognition. For instance, to address the challenges that RGB images fail to recognize white and transparent foreign fibers in seed cotton and traditional models yield low accuracy in identifying multi-size mixed impurities, Yunlong Zhang et al. [19] proposed a high-precision recognition method by combining hyperspectral imaging with their self-developed MJHResNet, which effectively improves the detection accuracy of various foreign fibers. However, this method suffers from limitations such as a restricted dataset, lack of spatial-spectral feature fusion, and insufficient industrial lightweight optimization. Wu Nianyi et al. [20] proposed the SSFNet model, which achieves accurate classification of cotton yellow wilt disease based on spectral feature fusion, demonstrating that deep learning can effectively extract useful information for fine-grained cotton recognition. However, this method has limitations, such as high computational complexity, which prevents it from meeting real-time detection requirements—a critical shortcoming for practical on-site detection.

To further tackle the detection difficulties of low-saliency foreign matter such as thread and hair in cotton, lightweight and high-speed YOLO series models have been widely optimized and applied. As the latest version of the YOLO series, YOLOv11 exhibits outstanding performance in both detection accuracy and speed. Benefiting from its enhanced feature extraction ability and lightweight architecture, it has been increasingly applied in various small object detection tasks. For example, Su Zhiwei et al. [21] proposed the Mulch-YOLO model based on improved YOLOv11 for real-time mulch detection in seed cotton, which effectively improves detection accuracy and reduces model parameters, demonstrating that lightweight deep learning can be well applied to real-time detection of low-visual-saliency mulch in cotton. Du Ziheng et al. [22] proposed a lightweight cone yarn varieties recognition model YOLO FSLP based on improved YOLOv11n, which greatly reduces model parameters and computation through lightweight design and channel pruning while maintaining 99.5% mAP@_50_, demonstrating that lightweight deep learning is suitable for high precision and real time recognition of textile products. Nevertheless, few studies have applied improved YOLOv11 frameworks to the detection of small impurities in seed cotton. Existing improved models still lack sufficient consideration of the complex characteristics of seed cotton images, and there remains obvious room for optimization in balancing accuracy, speed, and robustness in real-world scenarios. This study aims to fill this research gap.

## 3. Methods

### 3.1. YOLO11 Algorithm

YOLOv11 is a next-generation object detection model from the YOLO series, released by Ultralytics in 2024. It is an enhanced version built upon YOLOv8, optimizing both the network architecture and training methods to achieve an excellent balance between inference speed, detection accuracy, and model compactness. This makes YOLOv11 particularly well-suited for applications in agricultural production, such as cottonseed impurity detection.

**Figure 1:**
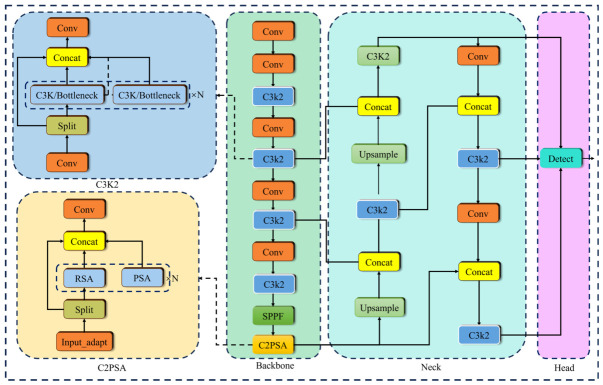
The YOLOv11 network structure.

YOLOv11 introduces several innovations in its design. The backbone network employs an improved version of CSPDarknet combined with a cross-stage partial network (CSPNet), effectively addressing issues like gradient vanishing and unnecessary computations, thus significantly enhancing feature extraction efficiency. The neck module adopts a Bi-directional Feature Pyramid Network (BiFPN), which flexibly integrates multi-scale features by utilizing bidirectional connections across different scales. This improves the transmission of fine-grained low-level details and high-level semantic information, both of which are crucial for detecting small impurities. In the detection head, YOLOv11 uses a decoupled, anchor-free approach, separating classification from regression. This reduces interference and eliminates the need for pre-defined anchor boxes, allowing it to better adapt to the size and distribution variations in cottonseed impurities. Figure 1 illustrates the YOLOv11 network structure.

### 3.2. YOLO11-SMS Algorithm

#### 3.2.1. Algorithm Improvement Strategy

**Figure 2:**
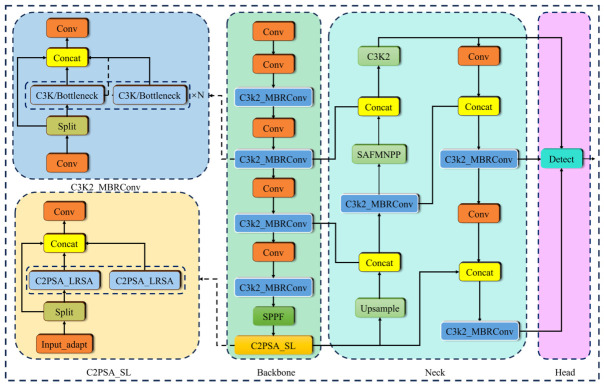
The network structure of the improved model.

This paper introduces three improvement strategies for the YOLOv11 model. First, the Local Self-Attention Mechanism (LRSA) [23] module is integrated into the Single-Head Self-Attention (SHSA) [24] module to design the C2PSA-SL module. This enhancement strengthens the model’s ability to learn local information while maintaining global context, thus improving both the efficiency and accuracy of object detection. Second, the Multi-Branch Reparameterized Convolution (MBRConv) [25] module is introduced, which enhances feature extraction capabilities while preserving the model’s lightweight nature. Finally, a Spatial Adaptive Modulation (SAFM) [26] module is added to the network head to improve the detection ability for small targets. Figure 2 shows the network structure of the improved model.

#### 3.2.2. C3K2_MBRConv

**Figure 3:**
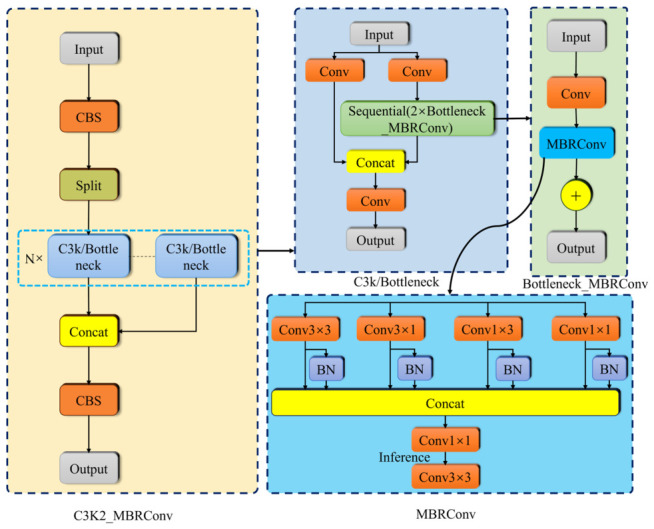
The network structure of the C3K2_MBRConv module.

When applying the YOLOv11n baseline model to cottonseed impurity detection, traditional single-branch convolution kernels present certain limitations in feature extraction. To address this issue, this paper proposes a Multi-Branch Reparameterized Convolution (MBRConv) module, designed to enhance the model’s ability to express features when processing different types of cottonseed impurities, such as cotton leaves, bolls, plastic film, and sand. The MBRConv module utilizes multiple branches with different types of convolutions to significantly increase feature learning capability. Additionally, it employs reparameterization techniques to reduce the impact of multiple branches on inference speed, ensuring that the entire network remains lightweight while adapting to various scenarios and meeting real-time detection requirements. Figure 3 illustrates the network structure of the C3K2_MBRConv module.

The traditional C3k2 module uses a single-branch convolution for feature extraction, which is simple but has a limited ability to represent complex textures and small targets, especially for impurities with varying shapes, such as cotton leaves, plastic film, and sand. In this paper, we propose a new C3K2_MBRConv module, which simplifies the core convolution part of the traditional C3k2 module.

The traditional single-branch convolution is replaced by a multi-branch reparameterized convolution (MBRConv), which utilizes four parallel convolutions (3 × 3, 1 × 1, 3 × 1, and 1 × 3) to achieve feature extraction in multiple directions and scales. Specifically, the 3 × 3 convolution serves as the main branch to capture fundamental spatial structures, textures, and contextual information for detecting small and complex-shaped impurities; the 3 × 1 and 1 × 3 asymmetric convolutions focus on directional edge and line features to capture fine structural details with fewer parameters and computations; the 1 × 1 convolution facilitates information exchange and combination across different feature channels. This combination achieves an optimal trade-off among multi-scale feature extraction, directional sensitivity, and lightweight design. Residual connections are strengthened to further enhance gradient propagation efficiency and preserve small target features.

Reparameterization techniques are used to decouple the training and inference structures, improving feature representation while ensuring that the inference time is not significantly increased, allowing the YOLOv11n model to remain lightweight.

Table 1 provides a more intuitive comparison between the C3K2_MBRConv module and the traditional C3K2 module.

#### 3.2.3. C2PSA_SL Module

Currently, the YOLOv11n baseline model has some limitations when applied to cottonseed impurity detection. Due to the fixed receptive field of the convolutional layers and the lack of effective feature attention guidance, the model struggles to balance local fine-grained features and global context information. This leads to the following issues in practical use: First, small impurities in cottonseed (such as sand particles and plastic film) are easily covered by cotton fibers and may not be detected. Second, the loose stacking of cottonseed often causes impurities to stick together or partially overlap, making it difficult to accurately determine the spatial relationship between impurities and cotton fibers when using only local features, which can result in incorrect classifications.

**Figure 4:**
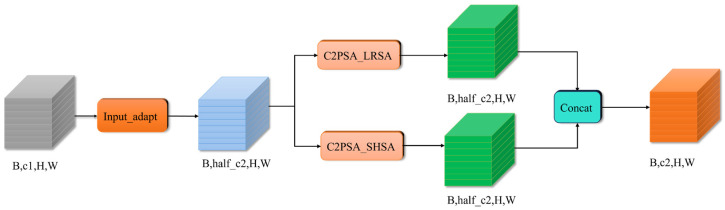
The framework structure of C2PSA_SL.

To address these problems, this paper integrates the Local Self-Attention C2PSA_LRSA into the Single-Head Self-Attention C2PSA_SHSA, creating a new module called C2PSA-SL. This module further extracts rich detail from local regions to enhance the model’s ability to recognize small target impurities. On the other hand, due to its global relational capability, the SL mechanism can focus on important local details while leveraging global context for better decision-making, improving small target feature extraction and localization. This significantly enhances the model’s robustness in complex cottonseed environments. The framework structure of C2PSA_SL is shown in Figure 4.

The C2PSA_SL module combines the C2PSA_LRSA and C2PSA_SHSA modules in parallel. First, the input feature tensor passes through a channel adaptation layer (Input_adapt), which adjusts the number of channels to half of the target output channels (half_c2 = c2//2) to ensure that the input channels for both branches are consistent. Then, the feature map is simultaneously fed into both the C2PSA_LRSA and C2PSA_SHSA modules, with both modules maintaining an output channel size of half_c2. Finally, the outputs from the two branches are concatenated along the channel dimension, resulting in a feature tensor with the original number of channels, c2.

(1) C2PSA_SHSA Module

**Figure 5:**
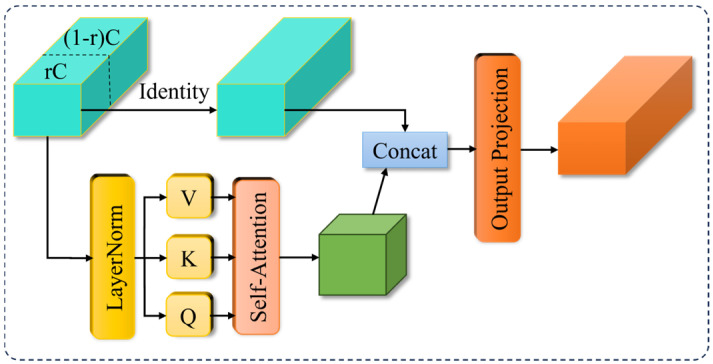
The structure of the SHSA single-head self-attention mechanism.

The C2PSA_SHSA module is formed by combining the Single-Head Self-Attention (SHSA) mechanism with C2PSA. In this module, the use of a single-head attention mechanism reduces computational complexity while simultaneously integrating both local and global information. This fusion enhances the detection speed and accuracy for small targets. Figure 5 illustrates the structure of the SHSA single-head self-attention mechanism.

First, the input feature channels are divided into two parts according to the ratio r. Here, r denotes the channel split ratio, which defines the proportion of input channels that will be processed by the subsequent self-attention operation. The split is performed contiguously along the channel dimension: specifically, the first rC channels are selected for attention processing, while the remaining (1−r)C channels are directly passed through. We empirically set the optimal channel split ratio to $r=\frac{1}{4}$ in our proposed model. One part contains rC channels, and the other part contains (1−r)C channels. The rC channels are used for the subsequent single-head self-attention layer operations, while the (1−r)C channels are directly passed through using an identity mapping to preserve the local detail features, as shown in Equation (1).

(1) $$X_{att},X_{res}=Split\left(X,\left[C_{p},C-C_{p}\right]\right)$$

In the equation, $C_{P}=rC$ and $C-C_{P}=(1-r)C$.

This ratio-based splitting design is adopted to reduce multi-head redundancy, lower memory access costs, and parallelly fuse global attention features and local detail features for better efficiency and accuracy. Ablation experiments verify that $r=\frac{1}{4}$ achieves the optimal trade-off between detection precision and inference speed.

Then, the rC portion of the channels undergoes Layer Normalization (LayerNorm). After that, the three vectors Q, K, and V are derived according to Equation (2).

(2) $${\tilde{X}}_{att}=Attention\left(X_{att}W^{Q},X_{att}W^{K},X_{att}W^{V}\right)$$

In the formula, $W^{Q}$, $W^{K}$, and $W^{Q}$ [27] are learnable linear projection weight matrices that transform $X_{att}$ into query (Q), key (K), and value (V) representations, respectively. This is a standard operation in self-attention mechanisms, as originally introduced in the Transformer architecture.

Afterwards, the self-attention mechanism in Equation (3) is applied to compute the global dependencies between features, resulting in global features weighted by attention.

(3) $$Attention\left(Q,K,V\right)=Softmax\left(QK^{T}/\sqrt{d_{qk}}\right)V$$

In the formula, $QK^{T}$ [27] denotes the matrix of dot-product compatibility scores between queries and keys, measuring the similarity between each query and all keys. $d_{qk}$ represents the dimension of the query and key, used to scale the scores and stabilize training.

Finally, the features obtained from the self-attention mechanism are concatenated with the (1−r)C portion of the channels. An output projection operation is then applied, which propagates the attention information across all channels, resulting in a feature tensor with the same dimensions as the input.

(4) $$SHSA\left(x\right)=Concat\left({\tilde{X}}_{att},X_{res}\right)W^{0}$$

**Figure 6:**
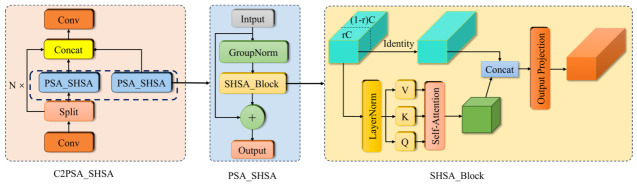
The network structure of the C2PSA_SHSA module.

To preserve the ability to learn spatial features while enhancing small target detection and reducing both computational complexity and parameter count, we introduce the SHSA single-head self-attention mechanism into C2PSA to create a more lightweight C2PSA_SHSA module. By replacing multi-head self-attention with single-head self-attention, not only can we reduce computation and the number of parameters, but we can also more effectively focus on the spatial features of important locations, thus stabilizing the extraction of detailed information, particularly for small targets. Figure 6 illustrates the network structure of the C2PSA_SHSA module.

(1) C2PSA_LRSA Module

**Figure 7:**
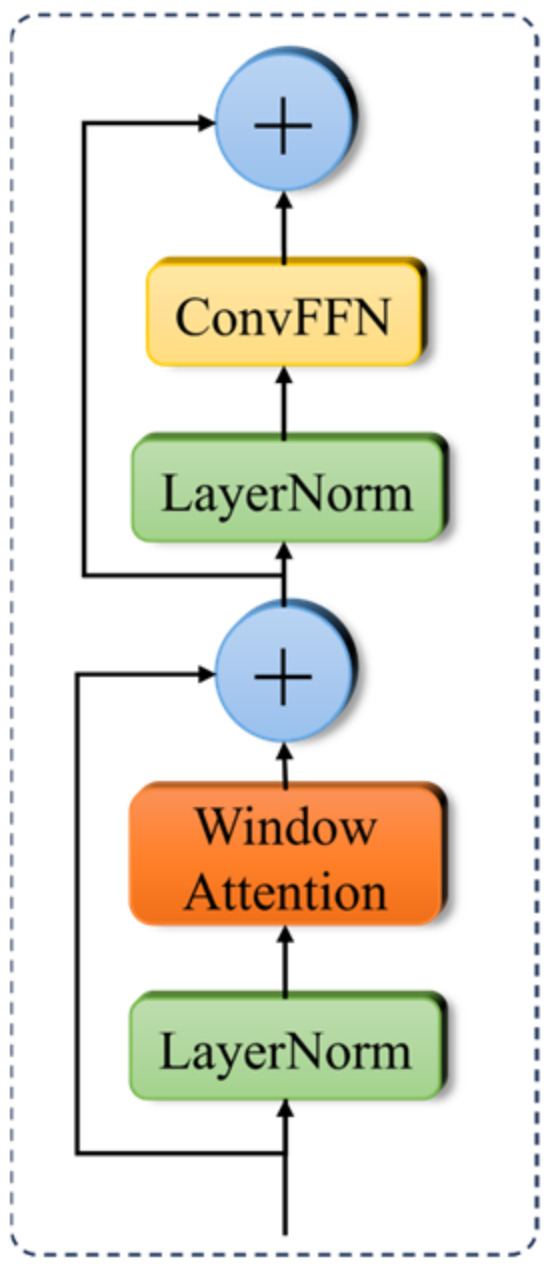
The structure of the LRSA module.

The C2PSA_LRSA module is proposed by integrating the Local Region Self-Attention Mechanism (LRSA) into the C2PSA framework. This module establishes attention connections between features at different positions within a local region, indicating the level of attention each position pays to other positions. This enables the network to focus more on important local information and effectively learn and utilize it. The structure is illustrated in Figure 7.

First, we apply a normalization operation to the input feature map to eliminate the size discrepancies between different channels, which helps achieve better results in the subsequent attention mechanism computations. After normalization, these feature maps are fed into the Window Attention module, where the feature map is divided into several small local patches. Each patch is projected using the same weight matrices $W^{Q}$, $W^{K}$, and $W^{Q}$ for Q, K, and V projections. Attention is computed within each local patch, allowing the model to effectively capture the relationships between local pixels.

Next, the feature map undergoes a residual connection merge, which helps prevent the loss of information during the recovery of local features. The merged feature map is then normalized again for the subsequent convolution operations. Finally, the ConvFFN convolution layer performs a dimensional expansion and reduction on the feature map, with the final residual connection ensuring smooth information flow. The specific expression is as follows:

(5) $$X_{out}=MSA\left(X_{0}W_{Q},X_{0}W_{K},X_{0}W_{V}\right)$$

In the formula, $W_{Q}$, $W_{K}$, and $W_{V}$ represent weight matrices, and MSA stands for multi-head self-attention operation.

**Figure 8:**
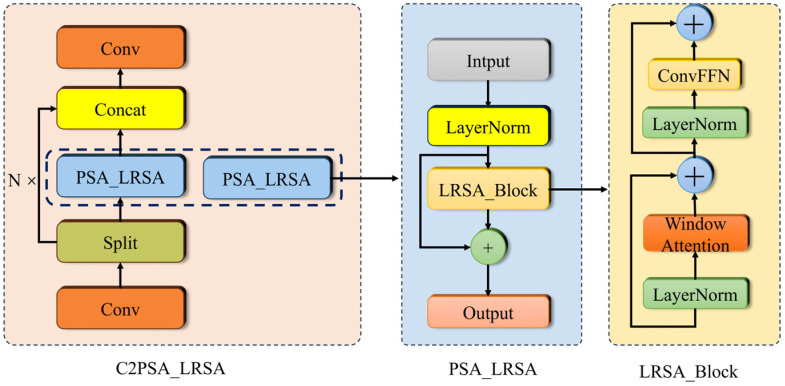
The structure of the C2PSA_LRSA module.

In object detection tasks, traditional convolutions are limited by the fixed receptive field, which makes it difficult to effectively extract small target edge textures and local details. While global self-attention can effectively learn relationships between different positions, its computational cost grows quadratically, making it unsuitable for fast detection requirements. To address these issues and further improve the modeling ability for channel, spatial, and local information while maintaining lightweight and fast processing, a new hybrid attention enhancement module, C2PSA_LRSA, is proposed. The structure of this module is illustrated in Figure 8.

First, a 1 × 1 convolution is applied to reduce the dimensionality, and the feature map is split into two branches: the main branch, which models multi-scale spatial features through multiple PSA_LRSA submodules (lightweight attention + ConvFFN + residual connections), and the direct connection branch, which retains local details through identity mapping. The features from both branches are then concatenated and passed through a 1 × 1 convolution to restore the channel count. Compared to the traditional C2PSA, this structure replaces multi-head self-attention with the lightweight LRSA, reducing computational complexity and enhancing inference speed while maintaining detection accuracy. Additionally, it exhibits better adaptability to high-resolution feature maps.

#### 3.2.4. SAFM Module

**Figure 9:**
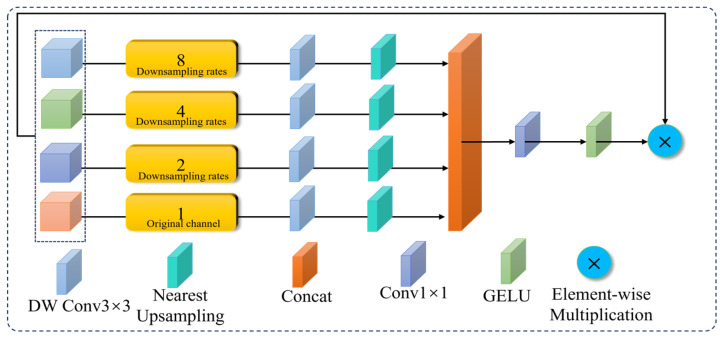
The structure of the SAFM module.

In cottonseed impurity detection, YOLOv11n has relatively weak feature extraction capabilities for small-sized impurities, such as hair and fine threads, often leading to missed detections due to background interference. To address this issue, this paper introduces the SAFM module, which operates during the feature fusion stage. With its advantages in multi-scale feature extraction and spatial adaptive modulation, SAFM effectively enhances the fine-grained feature representation of small target impurities, thereby improving the model’s accuracy in recognizing and detecting these small impurities. Figure 9 shows the structure of the SAFM module.

First, the input features are split into four groups of sub-features through channel splitting, namely: $\frac{1}{8}$, $\frac{1}{4}$, and $\frac{1}{2}$.

(6) $$\left[X_{0},X_{1},X_{2},X_{3}\right]=Split\left(X,dim=1\right)$$

The channel splitting operation uniformly divides the input feature into four consecutive, equal-proportion sub-features along the channel dimension (dim = 1). No random shuffling or arbitrary grouping is employed.

The first three groups are passed through adaptive max-pooling layers with downsampling rates of 8, 4, and 2, reducing the feature map to $\frac{1}{8}$, $\frac{1}{4}$, and $\frac{1}{2}$ of its original size, respectively. These scales are chosen to provide balanced multi-scale receptive fields while maintaining computational efficiency. As power-of-two factors, they align with hardware-optimized operations and fit the lightweight design constraints of our model. These downsampled features are then processed through 3 × 3 depthwise convolutions to extract features at different scales. Finally, nearest-neighbor upsampling is applied to restore the feature map to its original resolution. This process is represented by Equation (7).

(7) $$X_{i}={↑}_{p}\left(DW-{Conv}_{3\times 3}\left({↓}_{\frac{p}{2^{i}}}\left(X_{i}\right)\right)\right)$$

The last group is directly passed through a 3 × 3 depthwise convolution to extract the basic features. The four output feature groups are then concatenated along the channel dimension. Afterward, a 1 × 1 convolution is applied for channel compression and feature fusion, resulting in the fused features. This process is represented by Equation (8).

(8) $$X_{0}=DW-{Conv}_{3\times 3}\left(X_{0}\right)$$

Subsequently, the GELU activation function is applied to generate a spatial attention map, which is then element-wise multiplied with the original features to achieve dynamic spatial adaptive modulation. The final modulated features are obtained after this operation. This process can be represented by the following equation.

(9) $$X={Conv}_{1\times 1}\left(Concat\left[X_{0},X_{1},X_{2},X_{3}\right]\right)$$

**Figure 10:**
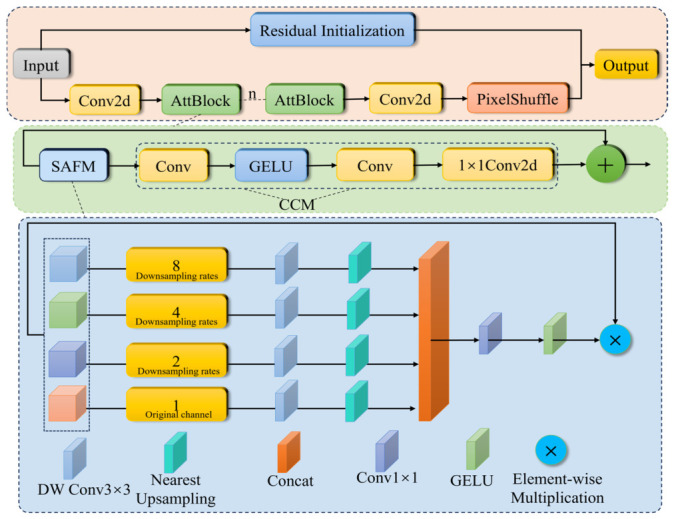
The network structure of SAFMNPP.

Based on the SAFM spatial adaptive feature modulation module, this paper designs a lightweight network, SAFMNPP, focused on enhancing small targets. By refining spatial feature modulation and retaining low-frequency information, it efficiently enhances small target features and recovers details. Figure 10 shows the network structure of SAFMNPP.

First, the input low-resolution image is processed in two parallel paths: one path directly performs residual initialization via bilinear interpolation to generate a base high-resolution image matching the target size; the other path first uses Conv2d to perform feature mapping (mapping the input channels to the model’s core dimension), followed by nAttBlock modules for lightweight spatial and channel feature enhancement. The feature map is then passed through Conv2d to expand the channel dimension, followed by pixel rearrangement (PixelShuffle) to convert the channel dimension information into spatial dimension information, thereby enlarging the image size. Finally, the base high-resolution image obtained from the residual initialization is element-wise added to the feature-enhanced image obtained from pixel rearrangement, resulting in the final high-resolution image output.

## 4. Experiments and Results

### 4.1. Experiments Environments

The experiments in this paper were conducted using the PyTorch 2.2.0 deep learning framework, with the operating system being Windows 10. The GPU used was an NVIDIA GeForce RTX 4060 with 8 GB of VRAM, and the CUDA version was 12.1. Python version 3.8.20 was used. In the experiment, the input image size was set to 640 × 640, the batch size was 16, the number of epochs was 170, and the number of workers was 4. The network model was trained from randomly initialized parameters.

### 4.2. Dataset

We captured samples of different types of cottonseed impurities using a visible-light camera, which allows for the acquisition of high-resolution spatial visual information consistent with natural human vision. This collection method provides clear insight into the morphological features and textural details of various cottonseed impurities, offering reliable data support for subsequent model detection. The dataset includes several common impurity types found during cottonseed processing, categorized into six types: paper, threads, films, hair, leaves, and ropes. The dataset is divided into training, validation, and test sets in an 8:1:1 ratio, with 1361 images in the training set, 171 images in the validation set, and 171 images in the test set.

In this study, we manually annotated all visible-light cottonseed impurity images using the LabelImg tool. Based on the spatial features of the images, we used rectangular bounding boxes to delineate the valid regions of each impurity target and assigned different colors to different types of impurities to clearly distinguish between the categories. Once the annotation was completed, we generated. TXT files that recorded the coordinates of each impurity target in the image, as well as some basic image parameters (such as spatial resolution, color channel range, pixel size, etc.), and the corresponding impurity class labels.

**Figure 11:**
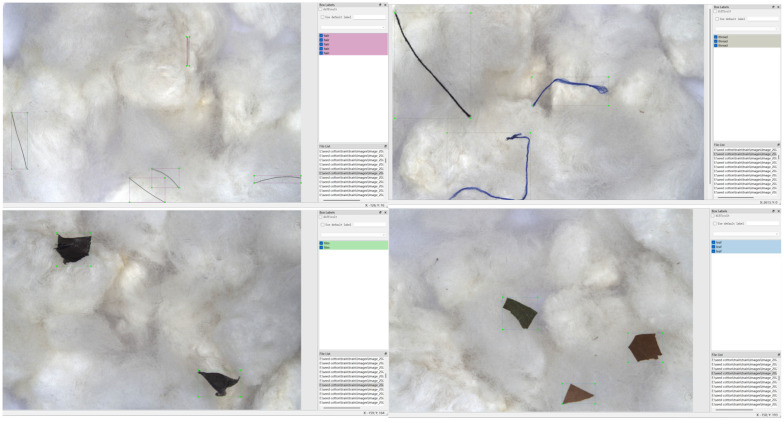
An example of cottonseed impurity annotations.

Considering the loose fiber structure of cottonseed, some impurities may be obscured or wrapped by cotton fibers, causing the boundaries of the targets to become blurred. To ensure the quality of the dataset, we only annotated impurities whose color features and spatial morphology were clearly visible. This approach ensures that the dataset provides reliable support for subsequent model training and detection. Figure 11 shows an example of cottonseed impurity annotations.

### 4.3. Evaluation Metric

To objectively and comprehensively assess the detection accuracy, localization precision, and inference efficiency of the visible-light cottonseed impurity detection model, the following metrics were selected in line with the practical requirements of object detection tasks:

(1) Precision (P): This represents the proportion of true positive impurity samples among all predicted positive samples. It reflects the model’s ability to minimize false positive detections.
  (10) $$P=\frac{TP}{TP+FP}$$
  where TP = True Positive (correctly detected impurities), FP = False Positive (non-impurities falsely detected as impurities).
(2) Recall (R): This represents the proportion of true positive impurity samples detected by the model among all actual impurity samples. It reflects the model’s ability to avoid missed detections (false negatives).
  (11) $$R=\frac{TP}{TP+FN}$$
  where FN = False Negative (impurities missed by the model).
(3) Mean Intersection over Union (mIOU): The arithmetic mean of the intersection over union (IoU) between predicted bounding boxes and ground truth boxes for all detected objects. It directly reflects the model’s spatial localization accuracy and is a core metric for measuring object detection performance.
(4) mAP@_50_: The average precision (AP) at an IoU threshold of 0.5, which evaluates the model’s performance at a relaxed localization precision.
(5) mAP@_50–95_: The average precision (AP) for all impurity categories across IoU thresholds from 0.5 to 0.95, stepping by 0.05. It comprehensively reflects the model’s detection performance at different localization precisions. mAP@_50_ focuses on evaluating the basic detection ability, while mAP@_50–95_ showcases the model’s robustness and high precision in detection.
(6) FPS (Frames Per Second): The number of visible-light cottonseed impurity image frames the model can process per unit of time. This metric includes the full process time, including image preprocessing, model inference, and non-maximum suppression. A higher FPS indicates better real-time detection performance, which is crucial for meeting the online detection requirements of real-world cottonseed processing scenarios.

The formulas for mAP@_50_ and mAP@_50–95_ are as follows:

(12) $$AP=\int_{0}^{1}P\left(r\right)dR$$

where *P*(*r*) is the precision at recall *r*, derived from the precision-recall curve.

(13) $${mAP@}_{50}=\frac{1}{N}\sum_{i=1}^{N}{AP}_{50i}$$

where *N* is the total number of impurity categories, and *AP*_50*i*_ is the average precision at IoU = 0.5 for category *i*.

(14) $${mAP@}_{50-95}=\frac{1}{N}\sum_{i=1}^{N}{AP}_{50-95i}$$

where *AP*_50–95*i*_ is the average precision at IoU between 0.5 and 0.95 for category *i*.

These metrics work together to provide a comprehensive evaluation of the model’s overall detection performance, including accuracy, robustness, and real-time capability.

### 4.4. Analysis of Improvement Results

Compared to the basic YOLOv11 model, the proposed YOLOv11-SMS model demonstrates significant improvements in detection accuracy and localization, as shown in the table. Specifically, precision (P) increased from 97.27% to 98.14%, reducing the error rate; mean Intersection over Union (mIOU) increased from 90.98% to 94.18%, improving the spatial localization accuracy of impurity targets; mAP@_50_ increased from 98.47% to 99.21%, maintaining good detection performance even with a large IoU threshold; and mAP@_50–95_ increased by 3.07 percentage points, from 79.42% to 82.49%. This indicates that the model has strong robustness for precise localization, effectively handling detection tasks even when impurity boundaries are unclear and features are complex.

Regarding inference efficiency, although the model’s FPS decreased slightly from 182.18 to 178.63, the speed of 178.63 FPS still far exceeds the industry standard (≥30 FPS), fully meeting the online detection requirements in cottonseed processing scenarios. Overall, YOLOv11-SMS achieves a better balance between accuracy and efficiency, making significant improvements in detection performance by slightly sacrificing speed. As shown in Table 2, the comparative data between YOLO11 and YOLO11-SMS is presented.

### 4.5. Ablation Experiments

To further verify the rationality of adopting $r=\frac{1}{4}$, ablation comparison experiments are carried out, and the detailed results are summarized in Table 3.

According to the ablation results in Table 3, $r=\frac{1}{8}$ yields faster inference frame rates but suffers from degraded mAP@_50–95_ detection accuracy. When $r=\frac{1}{2}$, both GPU and CPU inference FPS are drastically reduced, yet no positive accuracy gain is obtained. In comparison, $r=\frac{1}{4}$ achieves the optimal mAP@_50–95_ of 80.28%, while maintaining favorable and balanced real-time detection performance.

Accordingly, we select $r=\frac{1}{4}$ as the optimal channel split hyperparameter for the SHSA module. Meanwhile, all groups share consistent model parameters and FLOPs, which indicates that the channel ratio r only regulates the speed-accuracy trade-off and will not alter the inherent lightweight architecture and computational complexity of the proposed model.

To further demonstrate the effectiveness of each module, ablation experiments were conducted under the same parameter settings and training environment. The results of the ablation experiments are shown in Table 4.

As shown in Table 4, after introducing the SL module, the model’s mAP@_50–95_ increased from 79.42% to 80.47%, and FPS significantly improved to 230.76. This indicates that the SL module not only enhanced the feature extraction capability but also improved processing efficiency. After adding the SAFM module, the model’s precision (P) reached 98.56%, mIOU reached 93.83%, and mAP@_50–95_ reached 80.64%, demonstrating SAFM’s role in optimizing feature fusion and improving localization accuracy. As for the MBRConv module, it ensured that mAP@_50–95_ reached 80.82%, while also showcasing its adaptability in complex models through its lightweight design.

Further analysis of the module combinations shows that when the SL and SAFM modules are used together, the model’s mAP@_50–95_ increases further to 81.09%, with precision rising to 97.99%. This indicates that combining the attention mechanism with feature fusion modules leads to an additive effect on precision. Finally, after adding the MBRConv module, the final YOLOv11-SMS model increases mAP@_50–95_ to 82.49%, and mAP@_50_ also improves to 99.21%, which is a 0.74 percentage point increase compared to the baseline model.

Although the inference speed of the model decreased from 182.18 FPS to 178.63 FPS with the gradual integration of these modules, this is due to the increased computational load brought about by the synergy of the modules. Overall, the results of the ablation experiments demonstrate the necessity of the SL, SAFM, and MBRConv modules in the model. Their combination provides strong support for improving model performance while achieving a good balance between precision and efficiency.

To further analyze the contribution of each module to different impurity categories, Table 5 reports the per-class Average Precision (AP).

The MBRConv module significantly boosts multi-scale feature extraction through multi-branch reparameterized convolution. This results in an improvement in hair AP from 98.6% to 99.5% (+0.9 pp), thread AP from 96.0% to 98.8% (+0.8 pp), and leaf AP from 97.0% to 97.7% (+0.7 pp), illustrating its effectiveness in managing various impurity textures.

On the other hand, the C2PSA-SL module (abbreviated as SL) enhances the interaction between local and global features, leading to a marked increase in thread AP from 96.0% to 98.7% (+2.7 pp) and hair AP from 98.6% to 99.4% (+0.8 pp). This indicates its proficiency in capturing fine-grained details.

The SAFM module is tailored for improving the detection of small targets. As demonstrated in Table 4, when used independently, SAFM raises thread AP from 96.0% to 98.8% (+2.8 pp) and hair AP from 98.6% to 99.0% (+0.4 pp). These categories often involve fine, elongated, or small-area impurities, which can easily be overlooked in complex cotton fiber backgrounds. This improvement reinforces the idea that SAFM’s spatially adaptive feature modulation is effective in enhancing and preserving the discriminative features of small targets.

When all three modules are integrated (YOLOv11-SMS), the model achieves impressive AP scores across all categories, especially for paper (99.5%) and rope (99.5%), where intermediate configurations had previously shown lower AP. This highlights the combined benefits of SL, SAFM, and MBRConv, which not only improve small-target detection but also enhance the overall robustness and generalization of the model.

### 4.6. Comparative Experiments

To further highlight the performance advantages of the proposed method, comparison experiments were conducted with five models: YOLOv5 [28], YOLOv8 [29], YOLOv10 [30], YOLOv11 [31], and YOLOv12 [32]. The comparison experiment data are shown in Table 6.

**Figure 12:**
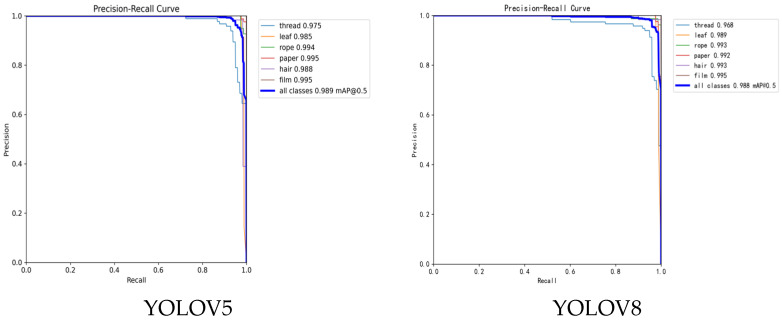

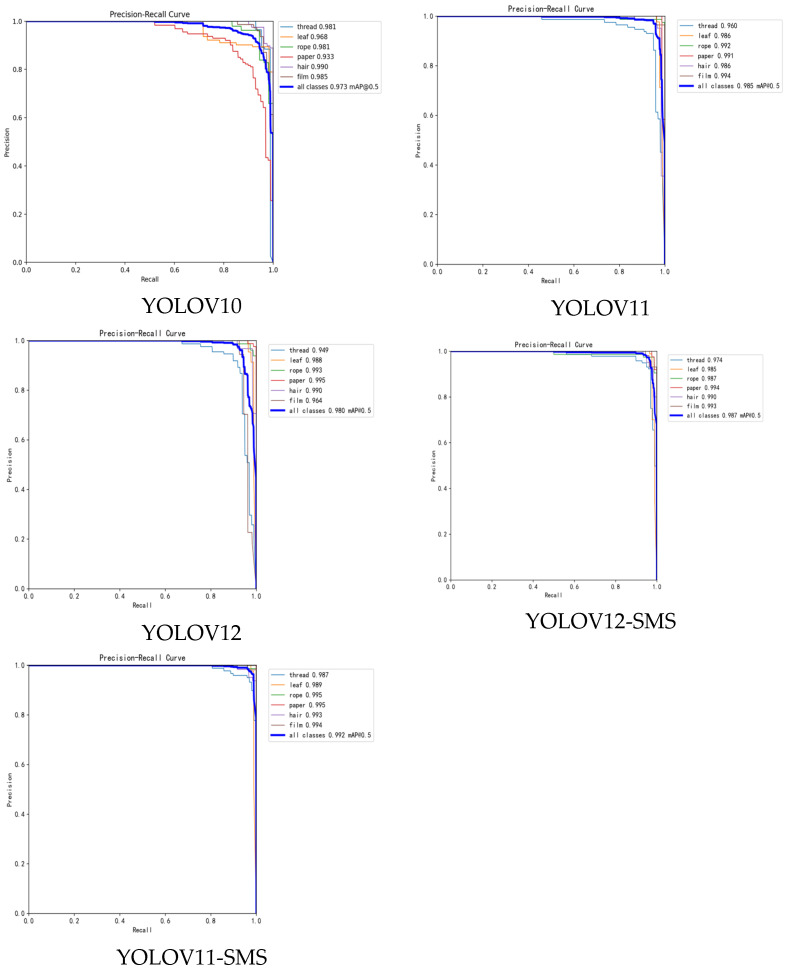
The P-R curve for each model.

The experimental results show that YOLOv11-SMS achieves superior overall performance while maintaining high accuracy. Compared to the baseline YOLOv11, its mAP@_50–95_ increased from 79.42% to 82.49%, with an improvement of 3.07 percentage points, demonstrating the effectiveness of the improved modules in complex backgrounds. Additionally, its mIOU reached 94.18%. Although the inference speed slightly decreased compared to YOLOv11, the model’s performance at 178.63 FPS still outperforms YOLOv5 (153.80 FPS) and other models, highlighting its excellent real-time performance and practical engineering value. Figure 12 shows the P-R curve for each model.

Compared with the equally improved YOLOv12-SMS model, YOLOv11-SMS exhibits clear superiority in both detection accuracy and real-time performance, achieving a far better balance for practical applications. Although YOLOv12-SMS improves over its baseline YOLOv12 in several metrics, including precision (98.19%), mIOU (86.40%), and mAP@_50–95_ (79.57%), it still falls noticeably behind YOLOv11-SMS in all core comprehensive indicators. Specifically, YOLOv11-SMS outperforms YOLOv12-SMS in mIOU by 7.78 percentage points (94.18% vs. 86.40%), in mAP@_50–95_ by 2.92 percentage points (82.49% vs. 79.57%), and in recall by 2.09 percentage points (97.15% vs. 95.06%), demonstrating stronger robustness and higher detection reliability in complex backgrounds. Most critically, YOLOv11-SMS maintains an inference speed of 178.63 FPS, which is more than twice the 73.97 FPS of YOLOv12-SMS. This significant speed advantage, combined with its consistently higher accuracy, confirms that YOLOv11-SMS is better suited for engineering scenarios that demand both high precision and real-time processing capabilities. The P-R curve for each model further illustrates these clear performance differences across all tested algorithms.

The proposed SMS modules achieve optimal performance when integrated with the YOLOv11 architecture, likely due to its better compatibility with the multi-scale feature fusion mechanism compared to YOLOv12.

**Figure 13:**
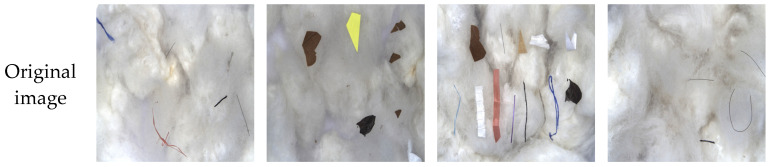

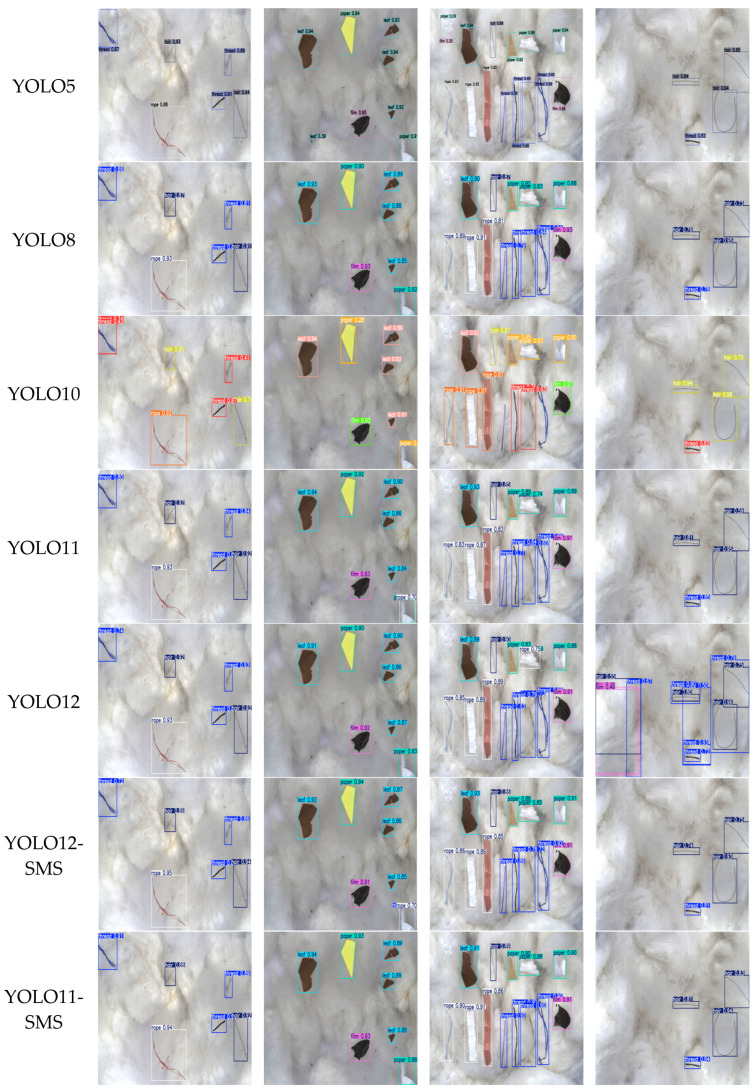
The inference results of the comparison models on different images.

**Figure 14:**
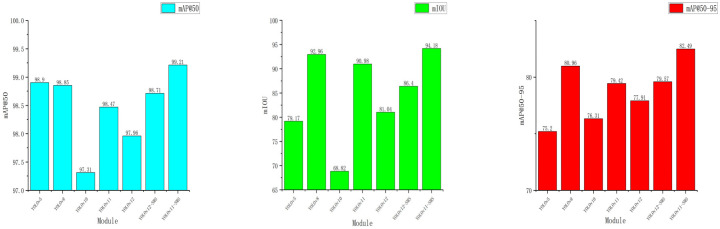
A comparison of mAP@**_50__–__95_**, mAP@_50_, and FPS for each model.

To more intuitively demonstrate the advantages of the proposed model, Figure 13 presents the inference results of the comparison models on different images. Figure 14 shows a comparison of mAP@_50–95_, mAP@_50_, and FPS for each model.

Through the inference images and comparison charts, we can more intuitively perceive the advantages of the proposed improved model.

## 5. Conclusions and Limitation

To address the challenges of detecting small targets and meeting real-time requirements in cottonseed impurity detection, this study proposes a new YOLOv11-SMS algorithm based on YOLOv11, incorporating spatial attention mechanisms, multi-branch reparameterization, and spatial adaptive modulation. Through the optimization of the network structure and a series of comparative experiments, the following key conclusions were drawn:

The proposed SMS composite enhancement strategy significantly improves the model’s ability to extract features from small impurities. The introduction of the spatial attention mechanism and adaptive feature modulation module enables the model to better detect small targets in complex backgrounds, thereby significantly enhancing detection accuracy.

The YOLOv11-SMS model demonstrates superior overall detection performance. Compared to the baseline YOLOv11 model, mAP@_50_ improved from 98.47% to 99.21%, and mAP@_50–95_ increased from 79.42% to 82.49%, confirming its clear advantage in cottonseed impurity detection tasks.

Overall, the YOLOv11-SMS algorithm proposed in this study effectively solves the problem of small target detection in cottonseed impurity detection and provides valuable insights for the detection of other related agricultural products.

Despite the promising performance of YOLOv11-SMS, several limitations remain to be addressed in future work.

First, the dataset used in this study has two main constraints: (a) it contains only six impurity categories (paper, threads, films, hair, leaves, and ropes) and is collected under controlled visible-light conditions, which may not fully represent the diversity of real-world cottonseed processing environments, such as varying illumination, humidity, and unexpected impurity types; (b) during annotation, only clearly visible impurities were labeled, and those heavily occluded by cotton fibers were excluded to ensure labeling quality. Consequently, the model has not learned to recognize severely occluded targets. These two factors together may limit the model’s generalization to complex, real-world scenarios where both impurity variety and occlusion levels are more challenging.

Second, although the model achieves high detection accuracy, the slight decrease in inference speed (from 182.18 FPS to 178.63 FPS) indicates a trade-off between computational complexity and real-time performance, which may affect deployment on resource-constrained edge devices.

Third, the proposed improvements are specifically tailored to the YOLOv11 architecture; their generalizability to other detection frameworks (such as Transformer-based detectors) has not been validated.

Addressing these limitations—by expanding the dataset with more categories, occluded samples, and diverse environments, as well as by exploring lighter or more transferable modules—will be the focus of our subsequent research.

## Figures and Tables

**Table 1:** Differences between C3K2_MBRConv and traditional C3K2.

Items	Traditional C3K2	C3K2_MBRConv
C3K = False	Standard Bottleneck	Standard Bottleneck
C3K = True	Bottleneck inside C3K module	Bottleneck_MBRConv inside C3K module
Convolution in Bottleneck	Single-branch 3 × 3 convolution	Multi-branch MBRConv3 convolution
Feature extraction	Single-scale	Multi-directional & multi-branch

**Table 2:** The comparative data between YOLO11 and YOLO11-SMS.

Module	P	R	mIOU	mAP@_50_	mAP@_50–95_	FPS
YOLOV11	97.27	96.38	90.98	98.47	79.42	182.18
YOLOV11-SMS	98.14	97.15	94.18	99.21	82.49	178.63

**Table 3:** Ablation analysis of channel split ratio r.

r	Params	FLOPs	GPU FPS	CPU FPS	mAP@_50_	mAP@_50–95_
1/8	2.56 M	3.21 G	74.4	5.0	98.89	80.06
1/4	2.56 M	3.21 G	59.4	4.8	98.72	80.28
1/2	2.56 M	3.21 G	38.3	2.5	98.64	80.06

**Table 4:** The results of the ablation experiments.

Module	P	R	mIOU	mAP@_50_	mAP@_50–95_	FPS
YOLOV11	97.27	96.38	90.98	98.47	79.42	182.18
YOLOV11 + SL	97.97	95.97	90.90	98.86	80.47	230.76
YOLOV11 + SAFM	98.56	97.12	93.83	98.87	80.64	214.20
YOLOV11 + MBRConv	97.55	96.61	91.27	98.88	80.82	193.39
YOLOV11 + SL + SAFM	97.99	95.53	92.25	98.69	81.09	189.16
YOLOV11 + SL + SAFM + MBRConv	98.14	97.15	94.18	99.21	82.49	178.63

**Table 5:** Class-wise AP comparison results of each proposed module ablation.

Module	Thread	Leaf	Rope	Paper	Hair	Film
YOLOV11	96.0	98.6	99.2	99.1	98.6	99.4
YOLOV11 + SL	98.7	99.1	99.4	97.2	99.4	99.2
YOLOV11 + SAFM	98.8	99.2	99.5	97.3	99.0	99.3
YOLOV11 + MBRConv	98.8	99.3	99.5	96.9	99.5	99.3
YOLOV11 + SL + SAFM	98.8	99.3	99.4	96.5	99.4	98.7
YOLOV11 + SL + SAFM + MBRConv	98.7	98.9	99.5	99.5	99.3	99.4

**Table 6:** The comparison experiment data.

Module	P	R	mIOU	mAP@_50_	mAP@_50–95_	FPS
YOLOV5	98.00	96.30	79.17	98.90	75.20	153.80
YOLOV8	97.90	97.11	92.96	98.85	80.96	174.95
YOLOV10	94.50	89.31	68.82	97.31	76.31	65.52
YOLOV11	97.27	96.38	90.98	98.47	79.42	182.18
YOLOV12	96.16	95.41	81.04	97.96	77.91	165.49
YOLOV12-SMS	98.19	95.06	86.40	98.71	79.57	73.97
YOLOV11-SMS	98.14	97.15	94.18	99.21	82.49	178.63

## Data Availability

The data used in this study are not publicly available because of relevant research project restrictions, but are available from the corresponding author upon reasonable request.
